# Gene Expression of Pneumocystis murina after Treatment with Anidulafungin Results in Strong Signals for Sexual Reproduction, Cell Wall Integrity, and Cell Cycle Arrest, Indicating a Requirement for Ascus Formation for Proliferation

**DOI:** 10.1128/AAC.02513-17

**Published:** 2018-04-26

**Authors:** Melanie T. Cushion, Alan Ashbaugh, Keeley Hendrix, Michael J. Linke, Nikeya Tisdale, Steven G. Sayson, Aleksey Porollo

**Affiliations:** a Department of Internal Medicine, University of Cincinnati College of Medicine, Cincinnati, Ohio, USA; b The Veterans Affairs Medical Center, Cincinnati, Ohio, USA; c Center for Autoimmune Genomics and Etiology, Cincinnati Children's Hospital Medical Center, Cincinnati, Ohio, USA; d Division of Biomedical Informatics, Cincinnati Children's Hospital Medical Center, Cincinnati, Ohio, USA; e Department of Pediatrics, University of Cincinnati College of Medicine, Cincinnati, Ohio, USA

**Keywords:** AIDS related, Pneumocystis, Pneumocystis pneumonia, anidulafungin, antifungal agents, ascus, echinocandin, opportunistic fungi, sexual development

## Abstract

The echinocandins are a class of antifungal agents that target β-1,3-d-glucan (BG) biosynthesis. In the ascigerous Pneumocystis species, treatment with these drugs depletes the ascus life cycle stage, which contains BG, but large numbers of forms which do not express BG remain in the infected lungs. In the present study, the gene expression profiles of Pneumocystis murina were compared between infected, untreated mice and mice treated with anidulafungin for 2 weeks to understand the metabolism of the persisting forms. Almost 80 genes were significantly up- or downregulated. Like other fungi exposed to echinocandins, genes associated with sexual replication, cell wall integrity, cell cycle arrest, and stress comprised the strongest upregulated signals in P. murina from the treated mice. The upregulation of the P. murina β-1,3-d-glucan endohydrolase and endo-1,3-glucanase was notable and may explain the disappearance of the existing asci in the lungs of treated mice since both enzymes can degrade BG. The biochemical measurement of BG in the lungs of treated mice and fluorescence microscopy with an anti-BG antibody supported the loss of BG. Downregulated signals included genes involved in cell replication, genome stability, and ribosomal biogenesis and function and the Pneumocystis-specific genes encoding the major surface glycoproteins (Msg). These studies suggest that P. murina attempted to undergo sexual replication in response to a stressed environment and was halted in any type of proliferative cycle, likely due to a lack of BG. Asci appear to be a required part of the life cycle stage of Pneumocystis, and BG may be needed to facilitate progression through the life cycle via sexual replication.

## INTRODUCTION

The echinocandins are a relatively new class of antifungal agents that are semisynthetic hexapeptides and have successfully been used to treat medically significant fungal infections caused by Aspergillus fumigatus and Candida albicans, with current use as a front-line therapy for invasive candidiasis (1, 2). The therapeutic potential of the commercially available agents caspofungin, anidulafungin, and micafungin for Pneumocystis pneumonia (PCP) is reduced due to lack of activity of these β-1,3-d-glucan (BG) synthase inhibitors on the trophic and other non-BG-expressing forms of its life cycle, which we showed to remain in large numbers after 3 weeks of treatment with up to 10 mg/kg of body weight echinocandin in a mouse model of Pneumocystis pneumonia (PCP) (3). Notably, these lingering forms did not appear to replicate and exhibited aberrant morphology. However, upon withdrawal of anidulafungin therapy, BG-containing asci reappeared within 2 weeks and continued to increase as the pneumonia returned.

Constant surveillance of the external environment is essential for the survival of yeast and fungi and stress responses to address insults such as osmotic shock or cell wall damage, including remodeling of cell wall structures and changes in cell cycle progression, as well as upregulation of heat shock proteins, depending on the stressor (4). One well-studied pathway is the mitogen-activated protein (MAP) kinase HOG1, the cascade of which initiates a temporary cell cycle arrest in response to osmotic stress, allowing the fungus to adapt and repair damaged cell wall structures. In Saccharomyces cerevisiae, progression through the cell cycle with damaged cell membranes results in cell lysis and death (4). To avert the process, DNA replication is halted in G_1_ by a glycogen synthase kinase 3 (GSK3)/Mck1-dependent degradation of Cdc6, a component of the prereplicative complex, in parallel with Sic1 stabilization (15). Cell wall damage induced by antifungal agents such as the echinocandins causes similar stress responses in pathogenic fungi. In Candida albicans, Cas5p has been identified as a pivotal biological responsive transcriptional regulator that facilitates the linking of cell cycle perturbations to cell wall stress caused by caspofungin (4). This single regulator was associated with over 60% of caspofungin-responsive genes, mediated by increased or decreased association with RNA polymerase II (Pol II). Like S. cerevisiae, cell cycle progression in caspofungin-treated C. albicans is arrested via Cas5p repression of Cdc6p, as well as members of the MCM complex (mini-chromosome maintenance) (4). In the filamentous fungi, transcriptome sequencing (RNA-seq) and network modeling in Aspergillus fumigatus after exposure to caspofungin revealed that genes involved in carbohydrate metabolism, such as those coding for the β-glucosidases and exo- and endo-β-1,3(4)-d-glucanases were differentially expressed, as they were in a previous study of Aspergillus niger (6, 85). In addition, the MAP kinase genes *sakA* (a *Hog1* orthologue) and *mpkA* were shown to interact with 15 and 6 genes, respectively, in a model of network cross talk in response to caspofungin, implicating the HOG and CWI (cell wall integrity) pathways (85). A genome-wide profiling of yeast exposed to three cell wall stressors, zymolyase, caspofungin, and Congo red was used to construct a common gene signature (7). A cluster of 43 genes were identified as a signature of cell wall maintenance (CWM) and included genes related to vesicular trafficking and transport, cell wall remodeling and morphogenesis, transcription and chromatin remodeling, signal transduction, and RNA metabolism. Thus, a stress response profile to caspofungin in yeast and fungi is emerging with common themes of cell wall remodeling, cell cycle arrest, and intracellular signaling.

As we have observed phenotypes in echinocandin-treated Pneumocystis murina similar to those described for fungi and yeast treated with echinocandins, we conducted an RNA-seq study to better understand these effects. Unique to the present study is the evaluation of P. murina in the mammalian host, and not in an *in vitro* setting, as in previous fungal studies. Gene expression profiles of P. murina after 2 weeks of treatment in immunosuppressed and infected mice were compared to those from untreated, immunosuppressed, and infected mice. Almost 80 P. murina genes were significantly up- or downregulated in the mice after 2 weeks of anidulafungin treatment. Gene signatures reported for fungi exposed to echinocandins were also found in the treated P. murina cells and included those for cell wall integrity, cell cycle perturbation, and notably, for sexual reproduction. This is the first study to report the effects of an echinocandin on global gene expression of a Pneumocystis species. The strong upregulation of genes related to sexual reproduction, cell wall integrity, and stress and the significant downregulation of ribosome function indicate that the fungi sensed their inhospitable environment and attempted, unsuccessfully, to initiate sexual reproduction. These findings also support the requirement of a sexual cycle and formation of asci, for progression through the full life cycle of Pneumocystis.

## RESULTS AND DISCUSSION

### Organism burdens before and after anidulafungin treatment.

**FIG 1:**
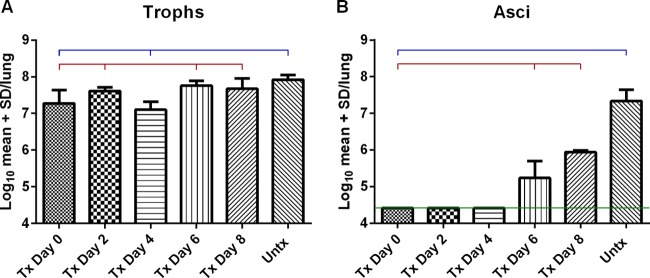
“Trophic” and ascus burdens pre-and postcessation of anidulafungin treatment. Two groups of mice were evaluated in this study. One group of P. murina-infected mice was treated with anidulafungin for 2 weeks, after which treatment ceased. The other group of mice was not treated. At the 2-week time point, 6 mice from the untreated group were sacrificed (“Untx”) and 6 mice that had been treated with anidulafungin were sacrificed (“Tx Day 0”). Mice were then sampled for organism burdens 2, 4, 6, and 8 days after ceasing the anidulafungin treatment (“Tx Day 2, 4, 6, or 8”). “Trophic” burdens are shown in panel A, and asci are shown in panel B. Blue bars indicate significant differences compared to the untreated mice at day 0; red bars indicate significant differences compared to the anidulafungin-treated mice at day 0. Significance was calculated using Newman-Keuls multiple-comparison test after calculation of ANOVA. Significance was accepted at *P* < 0.05. The green line in panel B denotes the level of detection by microscopic enumeration. No asci were observed in slides from these mouse lungs as log_10_ 4.2 is the level of microscopic detection. (At this time, we are unsure whether the forms left behind after anidulafungin treatment are all trophic forms, whether they include asci without cell walls or include transitional stages. Nonetheless, they do not express BG. Thus, the nuclei of all lingering forms were enumerated.)

The nuclei of non-BG-expressing “trophic” forms and asci were enumerated for all groups of mice in the study (Fig. 1). The nuclear burdens (titled “trophs” in Fig. 1) represented all life cycle stages that remained after anidulafungin treatment, as we suspect these lingering forms may not all be actual trophic forms. The numbers of these “trophic” forms (Fig. 1A) in mice after 2 weeks of anidulafungin treatment and before cessation of treatment (“Tx Day 0”) were significantly lower than those in the untreated mice at this time point (“Untx”), which is what we had observed in our earlier studies (3), suggesting an arrest in proliferation. However, after 2, 6, and 8 days postcessation, numbers of nuclei/trophic forms were not statistically different than those of the untreated mice (blue bar). The mice 4 days postcessation were the only group in which the trophic burdens were not statistically different from those in the treated mice at day 0 (red bar).

Anidulafungin, like all echinocandins, targets the ascus form of Pneumocystis. Therefore, it was not surprising that the treated mice had no detectable asci for up to 4 days after withdrawal of the anidulafungin (Fig. 1B). Asci were first detectable 6 days after withdrawal of anidulafungin, with increased numbers after 8 days of withdrawal. Both burdens were significantly different from those in the treated (Tx) mice at day 0 (red bar). All the treated groups had significantly fewer asci than the untreated group sacrificed on the day of anidulafungin cessation.

### Morphology of anidulafungin-treated P. murina.

In previous studies of the echinocandins, we showed that treatment with anidulafungin results in a loss of circumscribed ascus structures with anidulafungin, micafungin, and caspofungin (3). As a reference, new images from archival slides of that study are shown in Fig. S1 in the supplemental material. The clearly delineated cup-shaped asci in Fig. S1A represent the organisms in untreated mice, while amorphous groups of organisms characterized by diffuse methenamine staining, unclear borders, and the lack of asci from treated mice (1 mg/kg anidulafungin) are shown in Fig. S1B. Prominent in Fig. S1B are clear oval spaces (arrow) that suggest the previous presence of asci which have lost staining with methenamine silver.

**FIG 2:**
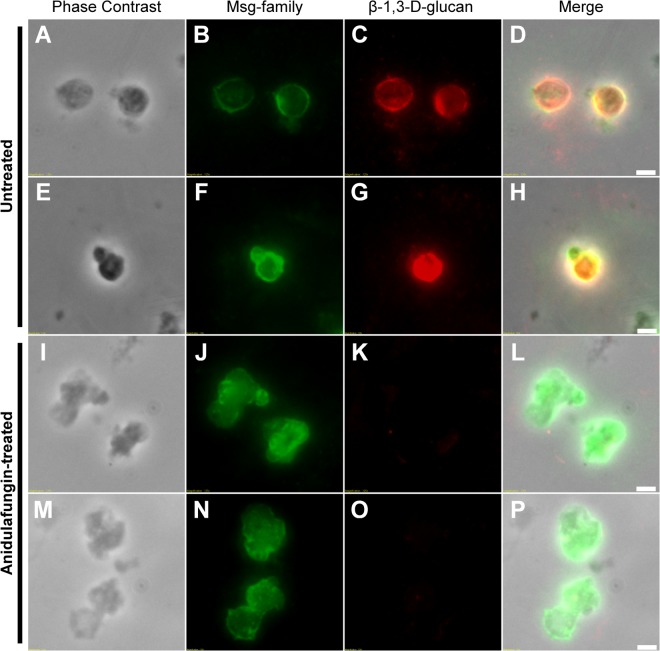
Fluorescent detection of β-1,3-d-glucan and Msg in P. murina cells from nontreated and anidulafungin-treated mice. Panels A through H are representative asci from untreated mice. Panels I through P are representative organisms from treated mice. Panels A and E show the morphology of P. murina cells observed under phase contrast in untreated, infected mice, while anidulafungin-treated P. murina cells displayed an atypical, nonspherical morphology (I and M). The 4 panels in each of the 4 representative groups are marked and include phase-contrast micrographs, reaction with an anti-Msg antibody, reaction with an anti-BG monoclonal antibody, and the merged panels. Detection of Msg family proteins was observed in both untreated (B and F) and anidulafungin-treated (J and N) P. murina cells. β-1,3-d-Glucan was detected in untreated P. murina cells (C and G) and shows coexpression with Msg family proteins (D and H). β-1,3-d-Glucan is notably absent or minimal in anidulafungin-treated P. murina cells (K and O). Note the aberrant morphologies of the treated organisms. Scale bars = 2 μm.

Untreated and anidulafungin-treated P. murina organisms from study 1 were stained with a monoclonal antibody (MAb) specific to β-1,3-d-glucan (red) and with polyclonal antiserum directed to the major surface glycoprotein (Msg) family (8) (green in Fig. 2). The Msg antiserum stains all developmental stages of Pneumocystis, while the anti-BG monoclonal would only be expected to stain the asci. Figure 2A to H show the phase-contrast images, Msg fluorescence, BG fluorescence, and a merged panel of representative asci from untreated mice. Notable is the robust staining with both BG antibody and Msg antiserum of the untreated organisms. In contrast, there was a dramatic loss of staining with the BG antibody of many of the treated P. murina cells (Fig. 2K and O). In addition, there was a lack of clearly circumscribed asci; instead, there were pleomorphic forms (Fig. 2J and N) that exhibited a more diffuse staining with the Msg antibody in the treated P. murina cells. Some treated organisms did retain some staining with the BG antibody, suggesting the breakdown of existing BG was a dynamic process (data not shown).

### Upregulated genes in anidulafungin-treated versus untreated P. murina.

The strongest signals in the 32 genes that were significantly upregulated in P. murina treated with the echinocandin for 2 weeks were those Schizosaccharomyces pombe and S. cerevisiae gene homologs associated with cell wall remodeling, specifically as these functions relate to cell replication and growth and responses to stress (Table 1). Most of the replication signals were associated with meiosis. An additional strong signal was found in genes without known homologs.

**TABLE 1:** P. murina genes significantly upregulated in the anidulafungin-treated mice

Function and GenBank ID	Gene product description	Expression (TPM)		FC (Tx0d/Untx)^*c*^	E value^*d*^	Identity, organism, or HP^*e*^
		UnTx^*a*^	Tx0d^*b*^			
Meiosis and cell wall integrity						
EMR11603.1	Gas4/5p	9.266	100.427	10.838	1e−128/8e−121	49%/50%, Sp
EMR10528.1	Mitogen-activated kinase kinase MKK2 (Pek1)	8.969	24.607	2.743	4e−113	52%, Sp
EMR10901.2	Glucan-1,3-β-glucosidase; glycosyl hydrolase family 17	76.321	203.799	2.67	8e−67	38%, Sc
EMR11636.1	Flavin carrier partial (FLC2)	46.014	117.458	2.553	3e−134	37%, Sc
EMR10161.1	GPI-anchored cell surface protein (Meu10)	281.502	653.933	2.323	9e−50	33%, Sp
EMR08655.1	Lsp1 (sphingolipid long-chain base-responsive)	56.259	127.557	2.267	3e−129	64%, Sc
EMR09455.1	Meu14 (eisosome component PIL1 domain)	100.218	201.691	2.013	4e−63	43%, Sp
EMR10198.1	Meu5/Crp79	81.402	155.74	1.913	2e−31	59%, Sp
EMR10169.1	Meiotic recombination (Dmc1)	324.034	599.66	1.851	0.0	73%, Sp
EMR09212.1	Bni4	76.109	135.674	1.783	2e−13	55%, Sc
EMR09078.1	Endo-1,3-β-glucanase (Eng1)	814.065	1,404.655	1.725	1e−09	47%, Sp
EMR10125.1	Ras guanine-nucleotide exchange (CDC25)	95.075	154.987	1.63	3e−50	32%, Sc
EMR09642.1	Meiosis specific coiled-coil (Mcp7)	144.307	233.293	1.617	1e−40	59%, Sp
EMR10540.1	Ubiquitin ligase complex F-box (GRR1)	41.962	65.299	1.556	9e−108	40%, Sc
Stress response						
EMR09987.1	Hsp16	526.759	1,574.851	2.99	1e−12	33%, Sp
EMR09253.1	Hsp72 (Ssa1/2)	2,743.877	5,897.959	2.149	0.0	80%, Sp
EMR09660.1	*myo*-Inositol transporter	16.314	34.202	2.097	0.0	90%, Pc
EMR08493.1	Hsp90	5,412.202	11,260.631	2.081	0.0	74%, Sp
EMR10428.1	CDP-diacylglycerol-glycerol-3-phosphate 3-phosphatidyltransferase	172.565	294.883	1.709	5e−39	44%, Sp
EMR09745.1	ARM repeat-containing; Pumilio superfamily	126.501	265.395	2.098	6e−114	55%, Sp
EMR09741.1	Mcp2; Pumilio family RNA binding repeat	81.233	153.703	1.892	2e−156	59%, Sp
EMR10181.1	rRNA processing Ipi1	103.609	249.864	2.412	5e−17	30%, Sp
Unknown function						
EMR09311.1	Hypothetical protein PNEG_02270	213.783	1,089.16	5.095	1e−38	60%, HP T552_02826 (Pc)
EMR08155.1	Hypothetical protein PNEG_03592	13.26	62.25	4.695	4e−135	58%, HP T552_02796 (Pc)
EMR09073.1	Hypothetical protein PNEG_02419	7.402	34.202	4.62	9e−126	56%, HP T552_02796 (Pc)
EMR09370.1	Hypothetical protein PNEG_02319	1,507.56	6,663.202	4.42	3e−92	60%, HP T552_02875 (Pc)
EMR10527.1	Hypothetical protein PNEG_01236	25.974	89.449	3.444	1e−115	92%, HP T552_04055 (Pc)
EMR08929.1	Hypothetical protein PNEG_02710	36.303	98.36	2.709	4e−132	67%, HP T552_01745 (Pc)
EMR10088.1	Hypothetical protein PNEG_01837	504.601	1,088.405	2.157	1e−56	81%, HP T552_03116 (Pc)
EMR08220.1	Hypothetical protein PNEG_03389	96.136	180.268	1.875	0.0	76%, HP T552_03364 (Pc)
EMR11410.1	Hypothetical protein PNEG_00433	298.172	556.408	1.866	3e−77	91%, HP T552_00422 (Pc)
EMR09148.1	Hypothetical protein PNEG_02491	189.756	316.926	1.67	9e−127	84%, HP T552_02724 (Pc)

a UnTx, P. murina extracted from infected, untreated mice. Expression is shown in transcripts per million (TPM).

b Tx0d, P. murina extracted from infected mice treated with anidulafungin for 2 weeks. Expression is shown in TPM.

c FC, fold change in P. murina gene expression between infected, untreated mice and mice treated with anidulafungin for 2 weeks.

d E value of sequence homology to the known genes as reported by the NCBI SmartBLAST tool.

e Sequence identity (percentage) to the known genes and organisms. Organism abbreviations: Sp, Schizosaccharomyces pombe; Sc, Saccharomyces cerevisiae; Pc, Pneumocystis carinii. HP, hypothetical protein for which the organism (in parentheses) and identity are for the closest species not P. murina in origin.

### (i) Meiosis and cell remodeling-related genes.

Upregulated gene homologs included the *Gas4*/*5p* (EMR11603.1), which is one of the genes coding for the β-(1,3)-glucosyl-transferases of the glycoside hydrolase family 72 (GH72) that are thought to remodel structural β-(1,3)-glucans through chain elongation, and the β-glucan-1,3-glucosidase in glycosyl hydrolase family 17 (EMR10901.2), which degrade glycans to control cell wall rigidity to allow for cell division, cell separation, or sporulation (9).

There are 5 Gas proteins identified in yeast, Gas1p to Gas5p, which are associated with life cycle stage-specific expression (10). Gas1p and Gas5p are expressed during vegetative growth and repressed during sporulation, while Gas2p and Gas4p are associated with sporulation. Gas3p is weakly expressed during vegetative growth and increases during sporulation (10). The P. murina homolog shared close homology to both the Gas4p and Gas5p genes from S. pombe and was the most highly expressed gene. Treatment with the echinocandins inhibits ascus formation, while mostly sparing the trophic forms which are considered the vegetative forms of Pneumocystis. The treated fungi appear to be trying to enter sexual replication and sporulation, as evidenced by the meiosis-specific gene homologs in Table 1, and thus a function more closely aligned with the sporulation-specific Gas4p seems more plausible.

The family GH17 of proteins are β-(1,3)-d-glucan endohydrolases and hydrolyze internal β-(1,3)-d-glucosidic linkages in polysaccharides, typically requiring a region of contiguous unbranched and unsubstituted β-(1,3)-d-glucosyl residues as the substrate. Many fungal cell wall hydrolases have chitinase or glucanase activity, and some of these enzymes also exhibit transglycosylase activity, permitting them to break and reform bonds within and between polymers, resulting in remodeling of the cell wall (11). Since Pneumocystis cell walls appear to lack chitin, it is tempting to postulate that one of the downstream effects by the echinocandins might be a catabolism of the existing BG via this enzyme and perhaps other glucanases. Since the echinocandins block the synthesis of β-1,3-d-glucan, the lack of formation of new asci would be expected; however, the preexisting asci should not be affected. Upregulation of the β-1,3-d-glucan endohydrolase may now explain the disappearance of the asci. Electron and light microscopic micrographs showed ablation of circumscribed asci after treatment with an echinocandin (12) (Fig. 2; Fig. S1). This GH17 putative protein appears to be distinct from the Eng1p reported previously, which was an endo-β-1,3-glucanase belonging to glycoside hydrolase family 81 and expressed only in the ascus form (75), and to the putative Eng2p, which was expressed in the trophic and ascus forms (13). In the present study, P. murina sequence EMR09078.1 shared significant homology with endo-β-1,3-glucanase (*eng1*), which facilitates cell separation in S. pombe. These proteins are associated with enzymatic cleavage of BG and the dissolution of the primary septum in fission yeast (14).

**FIG 3:**
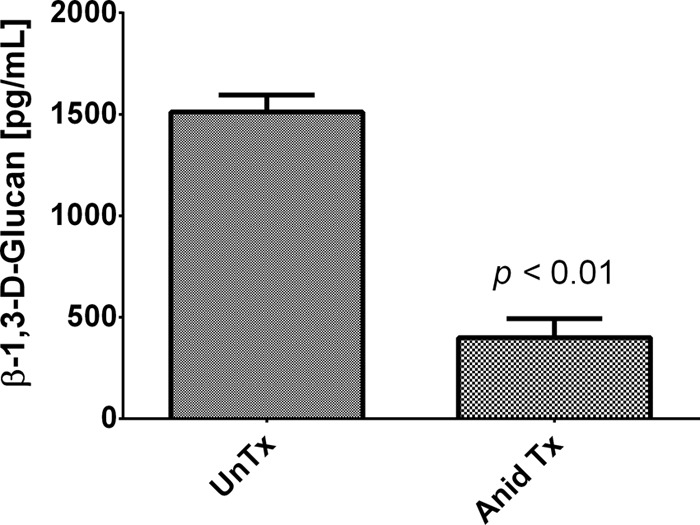
Measurement of β-1,3-d-glucan in the lungs of untreated versus anidulafungin-treated mice. β-1,3-d-Glucan contents in the lungs of untreated (UnTx) and anidulafungin-treated (Anid Tx [1 mg/kg anidulafungin for 2 weeks]) mice were quantified with the Glucatell kit. Significantly more of the intact polymer was present in the untreated (UnTx) versus treated (Anid Tx) mouse lungs (1,511.2 ± 166 versus 398.9 ± 187 pg/ml; *P* = 0.0001).

To provide further phenotypic evidence of BG degradation, we measured the amounts of BG in the lungs of untreated versus treated mice using the Glucatell assay and showed significantly more of the intact polymer in the untreated versus treated mouse lungs (Fig. 3).

In addition, a monoclonal antibody specific for β-1,3-d-glucan was reacted with P. murina extracted from the lungs of untreated and treated mice (Fig. 2). The stark contrasts between the organisms from the untreated mice versus treated mice are illustrated by the bright staining of the ascus walls compared to the diffuse staining of the treated organisms, supporting the contention that there is active degradation of extant BG. This downregulation of BG not only may serve as a signal for perturbation of sexual replication but could also contribute to the arrest of the cell cycle.

The MAP kinase Mkk2 (EMR10528.1) was shown in yeast to mediate the phosphorylation of the Mkc1 MAP kinase in response to cell wall assembly-interfering agents, such as zymolyase (which is a β-1,3-glucanase) (15). Such a response would be consistent with the effects of the echinocandin, as the lack of BG would specifically interfere with cell wall assembly, and this gene homolog would then be upregulated to repair the cell wall.

The hypothetical protein of P. murina (EMR11636.1) had most homology to S. cerevisiae flavin carrier protein 2. Yeast and Candida albicans both have 2 such homologs, Flc1 and Flc2, which have been reported to assist in the uptake of heme (16). The proteins encoded by the *flc* genes are located in the endoplasmic reticulum, and deletion of both resulted in phenotypes that suggest a role in cell wall integrity with loss of cell wall mannose phosphates and defects in cell wall assembly, which suggests they may play a role in the attempts to maintain cell wall integrity (16).

Several sequences shared homologies with genes associated with replication, both sexual and vegetative. The sequence of EMR10161.1 had homologies to the meiosis/sporulation-specific glycosylphosphatidylinositol (GPI)-anchored *meu10* of S. pombe, S. pombe and S. cerevisiae *ecm33*, and *sps2* of S. cerevisiae, which are involved in ascospore maturation, suggesting the treated organisms are attempting to produce asci in this stressed environment. EMR10169.1 and EMR09642.1 shared homologies with proteins driving meiosis, Dmc1 and Mcp7. Dmc1 is a meiosis-specific recombinase that facilitates repair of double-strand breaks during meiosis, while Mcp7 functions in the putative recombinase cofactor complex (17). Mcp7 acts downstream of Dmc1 in fission yeast for recombination and sporulation (18). It is tempting to think that similar mechanisms are involved in the postulated sexual replication of Pneumocystis, where the asci contain 8 not 4 ascospores (19). The *Meu5* gene (also called *Crp79*) is a nonessential gene that encodes a ribosomal binding protein (RBP) containing 3 RNA recognition motifs that is specifically induced during meiosis (20). Meu5p targets genes involved in spore wall synthesis and in the degradation of ascus walls, with the endoglucanases Agn2 and Eng2, which might suggest that Meu5 operates in concert with the Eng1 β-1,3-d-glucanase of P. murina.

Both the *Pil1* (EMR09455.1) and *Lps1* (EMR08655.1) genes were upregulated in response to anidulafungin treatment. The *Meu14*-encoded protein in fission yeast (Pil1) is involved in synthesis of forespore membrane, which is localized to the cytoplasm of the mother cell and ensures partitioning of gametic nuclei during meiosis II, a process essential for ascospore formation (76). It is also associated with remodeling of the plasma membrane as a major component of the eisosome complex (21). Eisosomes are structures that constitute the nanoscale furrow-like invaginations in the plasma membrane that harbor lipids, transporters, transmembrane proteins, and signaling proteins (22). The known eisosome components include the Pil1 and Lsp1 proteins as major components, with Pil1 being the primary organizer of these structures.

The *BNI4* gene (EMR09212.1) encodes an adaptor protein in yeast that enables bud neck assembly and might play a similar role in the separation of the vegetative cells of Pneumocystis (23). In S. pombe, cytokinesis proceeds after assembly and contraction of an actomyosin ring, followed by cleavage of the septum. The septum has a 3-layer structure with a primary septum, comprised mainly of BG surrounded on both sides by 2 secondary septa, composed of β-1,6-branched β-1,3-glucan and β-1,6-glucan. Although the gene encoding β-1,6-glucan synthase in P. carinii was identified (*kre6*), and this glucan was isolated from the cell wall as a minor component (77), no genes associated with it were differentially regulated with anidulafungin treatment. Cell separation requires dissolution of the primary septum for separation of the 2 daughter cells, after which the primary septum undergoes rapid degradation. Many genes are implicated in this process, especially the endo-β-1,3-glucanase (*eng1*) gene, which we found to be upregulated in response to anidulafungin. The sequence homology of EMR10540.1 to the E3 ligase *grr1* in yeast includes potential roles in morphogenesis, environmental response, and filamentous growth (24). The upregulation of these signals may indicate that the P. murina cells in the treated mice are trying, but unable, to undergo normal division or separation as they try to replicate.

An interesting homolog to EMR10125.1, *cdc25*, may be a potentially unique signal produced by the anidulafungin-treated P. murina. A recent study reported Cdc25p as the lynchpin for the mechanism of size control in fission yeast (25). Cdc25p is a mitotic activator with control of its transcript levels differentially regulated in smaller cells, which express less of Cdc25p, while larger cells express more. As the transcript levels increase, a threshold is tripped, which then signals cell division. Cdc25p dephosphorylates Cdc2p, permitting cell cycle progression through G_2_/M (26). Cell cycle arrest in response to environmental stress is dependent on Srk1p-mediated Cdc25p phosphorylation and nuclear export. However, downregulation of *srk1* (Table 2) suggests that this molecular signaling pathway that delays cell cycle progression in response to environmental or genotoxic insults, necessary for genomic stability, is being short-circuited. The upregulation of *cdc25* may result in the large or dysregulated cell morphology observed in the treated P. murina.

**TABLE 2:** Genes significantly downregulated in the anidulafungin-treated mice

Function and GenBank ID	Gene product description	Expression (TPM)		FC (Untx/Tx0d)^*c*^	E value^*d*^	Identity, organism, or HP^*e*^
		UnTx^*a*^	Tx0d^*b*^			
Meiosis and cell replication						
EMR10067.1	COP9 signalosome subunit 6 (Rpn8)	8.374	1.799	4.656	4e−08	30%, Sc
EMR11067.1	Adg3	6,110.184	1,861.178	3.283	2e−24	31%, Sp
EMR08305.1	Pheromone P-factor receptor (Mam2)	189.23	63.294	2.99	3e−42	33%, Sp
EMR08572.1	Pheromone-regulated multi-spanning membrane (Prm1)	299	130.057	2.299	6e−71	30%, Sp
EMR08216.1	Zds1	41.528	19.808	2.097	2e−05	51%, Sp
EMR08052.1	Calcium/calmodulin-dependent kinase (Srk1)	180.769	101.829	1.775	2e−148	54%, Sp
EMR08743.1	Single-stranded DNA binding protein (Ssb2)	58.526	35.555	1.646	3e−35	37%, Sp
Mitochondrial function						
EMR09040.1	Mitochondrial endodeoxyribonuclease (Pnu1)	1,136.987	398.104	2.856	6e−90	47%, Sp
EMR10336.1	Mitochondrial import subunit (Tom22)	178.033	94.027	1.893	1e−26	40%, Sp
EMR09346.1	Cytochrome *b*_5_ reductase 4	115.2	66.119	1.742	2e−18	41%, Sp
EMR10048.1	Mitochondrial fission process 1	214.822	128.712	1.669	2e−14	32%, Hs
EMR11107.1	Succinyl-ligase subunit α	99.526	59.797	1.664	6e−147	71%, Sp
Genomic stability						
EMR10220.1	Elongation of fatty acids 3 (Elo3, Gns1/Sur4)	108.911	50.703	2.148	4e−106	51%, Sp
EMR08516.1	Cytoplasmic tRNA 2-thiolation 1 (Ctu1)	591.814	320.682	1.845	3e−122	66%, Sp
Cell surface glycoproteins						
EMR08269.1	Major surface glycoprotein, partial	3,717.198	1,834.282	2.027	1e−19	27%, Pj
EMR09388.1	β-1,4-Mannosyltransferase (Alg1)	123.64	62.336	1.983	5e−111	42%, Sp
EMR08267.1	Major surface glycoprotein type II (Msr)	9,403.617	4,921.901	1.911	9e−05	20%, Pc
EMR08268.1	Major surface glycoprotein, partial	1,820.35	979.567	1.858	1e−05	20%, Pj
EMR08272.1	Major surface glycoprotein, partial	1,425.251	784.158	1.818	4.5	20%, Pc
EMR10408.1	Pig-F (predicted)	116.727	70.473	1.656	7e−60	38%, Sp
Ribosomal biogenesis/function						
EMR09837.1	40S ribosomal S6-A	774.972	394.259	1.966	2e−149	100%, Pm
EMR11496.1	rRNA-exonuclease (Rrp17)	258.855	137.663	1.88	4e−08	46%, Sp
EMR08677.1	rRNA processing (Fcf1)	86.943	46.463	1.871	3e−77	61%, Sp
EMR10517.1	40S ribosomal S5 (S7)	1,048.419	574.435	1.825	4e−76	59%, Sp
EMR11019.1	Transcription elongation factor, Elf1 family	60.632	36.027	1.683	2e−28	62%, Sp
EMR09557.1	RNA-binding Tma20 (translational machinery-associated proteins)	338.497	204.081	1.659	9e−70	54%, Sp
EMR10537.1	40S ribosomal S3	491.484	321.795	1.527	1e−132	86%, Sp
EMR10369.1	60S ribosomal protein L32	512.000	315.392	1.623	4e−54	66%, Sp
Cellular homeostasis/cell wall integrity						
EMR10852.1	Sho1	886.514	440.195	2.014	7e−16	31%, Sc
EMR09915.1	Hal9	3,444.312	1,812.795	1.900	1e−04	41%, Sc
EMR08979.1	Proteasome maturation factor (UMP1)	375.847	201.411	1.866	2e−15	33%, Sc
EMR11819.1	Prefoldin, β subunit (Yke2)	940.315	520.950	1.805	6e−19	52%, Sc
Unknown function						
EMR10405.1	Hypothetical protein PNEG_01120	22.502	1.000	22.502	NM	
EMR11131.1	Hypothetical protein PNEG_00727	43.895	4.857	9.038	NM	
EMR11709.1	Hypothetical protein PNEG_00143	15.105	1.952	7.738	7e−24	89%, HP T552_01243 (Pc)
EMR09469.1	Hypothetical protein PNEG_02411	2,864.366	518.069	5.529	4e−118	63%, HP T552_02964 (Pc)
KTW32888.1	Hypothetical protein PNEG_04307	114.167	27.417	4.164	3e−28	69%, HP T552_01884 (Pc)
EMR08688.1	Hypothetical protein PNEG_02866	75,752.191	37,354.644	2.028	3e−30	71%, HP T552_01591 (Pc)
EMR10004.1	Armadillo-like helical	195.497	99.319	1.968	0.0	88%, HP T552_03192 (Pc)
EMR08272.1	Hypothetical protein PNEG_03437	1,425.251	784.158	1.818	9e−18	27%, HP T552_03404 (Pc)
EMR08697.1	Hypothetical protein PNEG_02875	274.945	151.587	1.814	1e−98	89%, HP T552_02354 (Pc)
EMR09087.1	P-loop containing nucleoside triphosphate hydrolase (function unassigned)	109.213	65.845	1.659	1e−91	56%, Pl
EMR08958.1	Haloacid dehalogenase-like hydrolase (HAD)-like	56.259	35.679	1.577	1e−65	46%, Sp
AFR90421.1	Hypothetical protein (mitochondrion)	11,593.271	7,563.824	1.572	4e–60	50%, YP_009186399.1

a UnTx, P. murina extracted from infected, untreated mice. Expression is shown in transcripts per million (TPM).

b Tx0d, P. murina extracted from infected mice treated with anidulafungin for 2 weeks. Expression is shown in TPM.

c FC, fold change in P. murina gene expression between infected, untreated mice and mice treated with anidulafungin for 2 weeks.

d E value of sequence homology to the known genes as reported by the NCBI SmartBLAST tool. NM, no matches with known proteins.

e Sequence identity (percentage) to the known genes and organisms. Organism abbreviations: Sc, Saccharomyces cerevisiae; Sp, Schizosaccharomyces pombe; Hs, Homo sapiens; Pj, Pneumocystis jirovecii; Pc, Pneumocystis carinii; Pm, Pneumocystis murina; Pl, Protomyces lactucaedebilis. HP, hypothetical protein for which the organism (in parentheses) and identity are for the closest species not P. murina in origin.

### (ii) Stress-related genes.

The second significant group of upregulated genes in the treated group involved various responses to stress. The presence of 3 heat shock proteins indicates that the P. murina cells in these treated mice were in a stressed environment. EMR09987.1 had highest homology to the S. pombe small heat shock protein 16, which was shown to overcome a specific growth suppression in response to viral protein R (Vpr) (27). EMR09253.1 was homologous to the yeast heat shock protein 70 (Hsp70) family of proteins, specifically Ssa1p of S. pombe, which was recently shown to respond to misfolded proteins during the stress response by targeting them for degradation via the ubiquitin-proteosome system (28). The Hsp90p chaperone homolog to EMR08493.1 is reported to regulate the activity and stability of about 10% of cellular genes (29). In other fungi, Hsp90p helps to cope with stress induced by drug exposure and has been implicated in drug resistance mechanisms (5). Together, these signals suggest a stressed organism population that is producing misfolded proteins and other signs of stress to which the internal environment is attempting to respond.

P. murina sequence EMR10428.1 shared homology to CDP-diacylglycerol-glycerol-3-phosphate 3-phosphatidyltransferase, which catalyzes the committed step in the cardiolipin (CL) biosynthetic pathway (30). Cardiolipin (CL) is the signature phospholipid of mitochondrial membranes. Cardiolipin and phosphatidylethanolamine play essential roles in maintaining mitochondrial morphology in yeast and in mitochondrial biogenesis (31). In cells under stress, proapoptotic proteins such as the Bax protein are activated and translocate from the cytosol to the surface of the mitochondria (32). This can lead to a cascade that releases more apoptotic factors, eventually resulting in apoptosis. Integral to the process is permeabilization of the mitochondrial outer membrane (MOM). Cardiolipin plays a critical role in this process by recruitment of Bax to the MOM, facilitating the protein's insertion into the membrane, resulting in further permeabilization to these oxidatively damaged membranes. The upregulation of CL appears then to be a response to the stressful treatment environment signaling the P. murina cells to undergo apoptosis.

Interestingly, starvation for inositol resulted in a 3-fold increase in CDP-diacylglycerol-glycerol-3-phosphate 3-phosphatidyltransferase expression in S. pombe (33). Like S. pombe, Pneumocystis species are *myo*-inositol auxotrophs, and the upregulation of the gene coding for this protein may indicate an increased need for this essential nutrient in this stressed environment. Supporting this contention would be the significant upregulation of the *myo*-inositol transporter homolog to the P. carinii gene (EMR09660.1), which we have previously shown to be the critical (and only) source for *myo*-inositol (78).

Two genes, EMR09745.1 and EMR09741.1, shared homology with other stress-related functions, the ARM RNA binding repeat-containing Pumilio superfamily genes (Table 1). Puf proteins repress mRNAs by recognition of sequences located in the 3′ untranslated region (UTR) of target mRNAs (79). Within the Puf proteins are a conserved RNA-binding domain comprised of 8 imperfect repeats of a 36-amino-acid sequence with short flanking regions. These 2 genes upregulated by P. murina shared homologies to the Puf4p gene in yeast and the RNA-binding repeat region. One way in which eukaryotic cells respond to stress is by regulating mRNA decay and translation via the Puf family of proteins (34). The Puf4p in yeast was found to act exclusively through stimulation of deadenylation leading to decay of message. Puf4 regulates HO endonuclease expression, which controls mating-type switching in yeast via recombination and may be involved in cell differentiation/fate.

EMR10181.1 was homologous to the rRNA processing gene *Ipi1*, which is associated with the *Grc3* gene in S. pombe that facilitates the processing of rRNA necessary for ribosome biogenesis and heterochromatin assembly (35). The increased expression of this gene is curious as most other ribosome-associated genes were downregulated (Table 2).

### Downregulated genes.

Genes that were downregulated in the anidulafungin-treated P. murina were more diverse in functional groupings than those that were upregulated. These genes encoded proteins that could be grouped in the general categories of cell replication, mitochondrial function, genome stability, cell surface glycoproteins, ribosomal biogenesis, and cell homeostasis (Table 2). The strongest downregulated genes were those with unknown function, much like the highest-upregulated genes (Table 1).

### (i) Meiosis and replication-related genes.

Within the strongest downregulated genes with known homologies was the homolog to the COP9 signalosome (CSN) subunit 6 (EMR10067.1). The eight subunits making up the CSN complex are highly conserved among eukaryotes and are associated with proteolysis, signal transduction, and cell cycle regulation. Interestingly, CSN is found in fission yeast, but not in S. cerevisiae (36). The aberrant expression of a single CSN subunit can shift the equilibrium of the whole-cell cycle progression. Specifically CSN6 (EMR10067.1) appears to be associated with G_2_/M progression, as silencing of the CSN6 gene with antisense sequences resulted in G_2_-phase arrest (36). If DNA damage occurs during the G_2_ phase, the G_2_ DNA damage checkpoint provides sufficient time for cells to repair the damage prior to nuclear and cellular division (37). Downregulation of this subunit may result in halted mitosis and genomic instability. It is then unclear if the remaining P. murina cells are actively undergoing mitosis at this point, as the organism burdens stay somewhat constant over treatment time (3).

EMR11067.1, a homolog to Adg3p in S. pombe, was shown to be under the control of the transcription factor Ace2p during the later phase of the mitotic replication cycle, which exerts its control in the separation of the cells (38). The downregulation of this homolog in P. murina may coincide with the lack of cleavage of some of the cells remaining in the lung after treatment and could explain, in part, some of the morphological changes in the treated P. murina cells as a failure to completely separate.

Of note, a homolog (EMR08305.1) to the S. pombe pheromone P-factor receptor gene *mam2* (*Ste2* in yeast) was significantly downregulated. This receptor's cognate pheromone, P-factor, is a simple peptide composed of 23 amino acids. In S. pombe, P cells (also called h+ cells) secrete P-factor, which activates the G-coupled receptor Mam2p on M cells, which initiates the mating reaction. Although it is curious that genes associated with meiosis were upregulated in the treated P. murina cells and that one of the receptors necessary to initiate the mating response would be downregulated, this downregulation could be interpreted as a result of the unsuccessful efforts for induction of sexual reproduction.

Another gene homolog that had a strong downregulation signal was *prm1* (EMR08572.1). In S. pombe, this gene's protein facilitates an early event of mating, the fusion of the 2 mating-type cells. A *prm1* mutation completely abrogated this event, leading to abnormalities in the plasma membrane and cell wall in the area of cell-to-cell interaction (39). Prm1p is only expressed during mating, is necessary for bilayer fusion of the mating pairs, and localizes to the surface of the mating pairs. If the mating pairs cannot proceed after this arrest, unfused mating pairs produce cytoplasmic bubbles made of one or the other plasma membrane pushed into the counterpart's cytoplasm. Lysis can then occur as a product of this defective membrane fusion. Treatment with anidulafungin results in highly aberrant morphologies of P. murina in mouse lungs (Fig. 2), suggesting that downregulation of this gene may contribute to the changed morphology observed in the mouse lungs or that sexual replication was not occurring.

A homolog to the *Zds1* gene was identified (EMR08216.1). Zds1 has been shown to be associated with many cellular roles in fission yeast, including sexual differentiation, calcium tolerance, cell wall integrity, viability, and cell morphology (40). A *zds1*Δ strain in fission yeast resulted in abnormal cell morphology that included irregularities in the secondary septum, which may also be indicative of the inability to proceed through replication.

*srk1* (Sty1-regulated kinase1) encodes a serine/threonine kinase homologous to mammalian calmodulin kinases in S. pombe (41) (EMR08052.1). Srk1p is a component of a complex with Sty1p, which directly phosphorylates it and upon stress is translocated from the cytoplasm to the nucleus. After nitrogen starvation, which typically initiates meiosis in S. pombe, *srk1* deletion mutants entered meiosis at an accelerated pace, which indicates that *srk1* functions as an inhibitor of meiosis and by its downregulation under anidulafungin pressure may then allow meiosis to proceed. On the other hand, *srk1* mutants had a higher percentage of septated cells, suggesting an increase of progression through mitosis (41). It may be postulated that in the absence of the ability to proceed into ascus production due to the lack of BG, the cells are then pushed toward septation as an alternative means of separation, if indeed any replication is occurring.

P. murina EMR08743.1 shared homology with the S. pombe SSb2 protein, considered a nonconventional heat shock protein, which was shown to play a minor role in glucose repression (42). With the exception of the last homolog, the signals in this group support the contention of dysregulation of replication.

### (ii) Mitochondrion-associated functions.

The endonuclease G homolog in S. pombe (*pnu1*) shared homology with a P. murina gene (EMR09040.1) that was downregulated after treatment with anidulafungin. These proteins are mitochondrial nonspecific nucleases that are highly conserved among eukaryotes. The enzyme is released from mitochondria and then digests nuclear DNA. In the budding and fission yeasts, this homolog was shown to trigger apoptosis. Deletion of the yeast gene (also called *Nuc1*) diminished apoptotic death when mitochondrial respiration was increased, but under conditions of oxidative phosphorylation repression, necrotic death was enhanced (43). This then implies that the expression of *pnu1* during respiration promotes apoptosis, while it protects from nonapoptotic death when it is suppressed.

The β-barrel protein Tom40 and the α-helically anchored membrane protein Tom22 (EMR10336.1) are conserved subunits of the protein translocase of the mitochondrial outer membrane (TOM). Yeast-type Tom22 functions as a secondary protein import receptor and is also required for the stability of the TOM complex (44). In the absence of Tom22, the complex dissociates into smaller Tom40-containing units, and the biogenesis of mitochondria is strongly impaired (45).

Cytochrome *b*_5_ reductases function in electron transfer in the mitochondria and in the endoplasmic reticulum. Microsomal cytochrome *b*_5_ reductases are involved in the biosynthesis of fatty acids and complex lipids, including sphingolipids and sterols. While S. cerevisiae contains a single gene encoding a cytochrome *b*_5_ reductase, deletion of the 3 genes encoding cytochrome *b*_5_ reductases in the phytopathogenic fungus Zynoseptoria tritici resulted in defects in disease progression and asexual sporulation (46). Whether the EMR09346.1 sequence reflects a true cytochrome *b*_5_ reductase is uncertain, since the homology was shared only at a very specific stretch of these genes carried by the fruit fly, mouse, human, zebrafish, and Caenorhabditis elegans.

A homolog to human DRP1 had the highest match to EMR10048.1, without any fungal homologs, including yeast homolog to DRP1, dynamin 1 (Dnm1). The dynamin-related protein 1 in humans is required for mitochondrial membrane fission (47). Altered mitochondrial morphology has been associated with an imbalance between fission and fusion processes leading to neurodegenerative diseases such as Alzheimer's and Parkinson's disease and, generally, with aging (48). In yeast, loss of function of the gene coding for this protein results in a “net” of interconnected tubules (49). Succinyl-ligase subunit α (EMR11107.1) is involved in the tricarboxylic acid cycle that synthesizes succinate from succinyl-coenzyme A (CoA) (ligase route).

The downregulation of these genes associated with mitochondrial function and biogenesis may indicate a general dysfunction of mitochondria in the stressed P. murina cells; however, a concomitant upregulation of enzymes associated with glycolysis was not observed, suggesting that mitochondrial respiration was still functional, albeit with some impairment.

### (iii) Genomic stability-related genes.

Very-long-chain fatty acids, typically containing 26 carbons, are synthesized from palmitoyl-CoA by microsomal fatty acid elongases (Elo1-3p) in yeast or Gns1p/Sur4p in S. pombe. P. murina possesses a homolog to the gene coding for a protein that is involved in the synthesis of very-long-chain fatty acids, Elo3, in yeast, which specifically catalyzes elongation from C_24_ to C_26_. Yeast sphingolipids mostly contain the C_26_-phytoceramide with hexacosanoic acid, which is the precursor for complex sphingolipids such as inositol phosphorylceramide, mannosylinositol phosphorylceramide, and mannosyldiinositol phosphorylceramide. Null mutations of the *elo3* gene perturb the synthesis of sphingolipids and phosphatidylinositol/inositol phosphate metabolism, which mediates signaling functions, regulation of longevity, senescence, and telomere length (50). Downregulation of this gene might predict dysregulation of sphingolipid metabolism concomitant with an accelerated but reduced life span. Alternatively, perturbation of telomeric function may have had an impact on the expression of the *Msg* genes, which are located at the telomeric ends of the chromosomes (51, 80) and which were downregulated in the anidulafungin-treated P. murina, as discussed below.

The uridine at the first anticodon position of the tRNA of glutamate, lysine, and glutamine is thiolated, which appears to be critical for proper decoding of the mRNA by restriction of wobble and efficient codon-anticodon interaction (52). The Ctu1p-Ctu2p (cytosolic thiouridylase) complex is responsible for the 2-thiolation in the fission yeast (52). Inactivation of this complex leads to a decrease of viability with notable ploidy abnormalities and aberrant development. Such a downregulation could lead to misreading and frameshifts with a generalized severe genome instability.

The signals in this class of putative proteins indicate that the treated P. murina cells are responding with generalized signals indicating genomic instability.

### (iv) Cell surface glycoprotein genes.

Four genes encoding the “major surface glycoproteins” (MSGs) of Pneumocystis were downregulated. This gene superfamily comprises up to 10% of Pneumocystis genomes. These genes are genetically similar, repetitive, located at the telomeric ends of chromosomes, and produce GPI-anchored glycoproteins with N-linked glycosylation (80). These glycoproteins migrate at approximately 120 kDa on reducing protein gels. The MSGs are the most abundant cell surface protein in these fungi and function in the interactions with host cells. Their ability to undergo antigenic variation may play a role in evasion of host immune responses (53, 81). These glycoproteins may also facilitate the tight interdigitation with the type I pneumocyte, to which they preferentially adhere in the mammalian alveolar spaces. In addition, the life cycle stages of Pneumocystis within the alveoli are closely adherent to one another in a biofilm-like matrix (82), and these surface glycoproteins could also play a role in facilitating the adherence of different mating types. It is striking that such a strong signal in their downregulation was apparent in the treated fungi, but explainable by the downregulation of ribosome biogenesis and subsequent protein synthesis discussed below. Alternatively, or in addition to, the downregulation of the homolog to *Elo-3*, which perturbs telomeric function as noted above, may have been complicit in the reduced expression of this family of genes. These data appear to be in contrast to the fluorescent staining with the anti-Msg antibody (Fig. 2), which shows some, though diffuse, staining of Msgp. However, it should be noted that this is a gene superfamily containing many members and the antibody is directed to common epitopes in the proteins. While some members may have been downregulated, others in the family may have been expressed.

Concomitant with the downregulation of the Pneumocystis-specific *Msg* genes, a homolog to *Alg1* was also downregulated. Yeast *Alg1* (asparagine-linked glycosylation 1) encodes a β-1,4-mannosyltransferase that adds the first mannose onto GlcNAc2-PP-Dol to produce a core trisaccharide, Man1GlcNAc2-PP-Dol. Little else is known about its specific roles, but it appears to be necessary for a variety of functions and thus required for viability (54). MSGs are mannose-containing glycoproteins with N-linked glycosylation sites, and the downregulation of Alg1p may interfere with posttranslational modification. Alternatively, the apparent global reduction in protein synthesis may have in turn downregulated this gene since Msgp synthesis was decreased.

Glycosylphosphatidylinositol (GPI) anchors may anchor eukaryotic cell surface proteins to the membrane. GPI is synthesized from phosphatidylinositol and attached to the C terminus of nascent proteins as a posttranslational modification. The homolog to EMR10408.1, *Pig-F*, functions to add ethanolamine phosphate to the third mannose, facilitating the attachment of GPI to proteins (55). Downregulation would infer a defect in the anchoring of surface proteins in the treated P. murina. The most abundant surface proteins are in the MSG family: the structure of some but not all members includes a signal peptide at a region called the upstream conserved sequence (UCS) and a hydrophobic tail at the C terminus that can function as a GPI anchor (56). Downregulation of the anchoring process may have in turn signaled a downregulation of Msgp expression, as was evident in Table 2. Alternatively, like the downregulation of *Alg1*, the reduction in protein synthesis concomitantly reduced the need for anchoring.

### (v) Ribosomal biogenesis and function.

Ribosomal biogenesis in eukaryotes is an evolutionarily conserved, tightly regulated, and complex process that involves hundreds of proteins and RNAs (57). The synthesis and processing occur in the nucleolus, where ribosomal DNAs (rDNAs) in tandemly repeated transcription units are transcribed by RNA polymerase 1 to precursor rRNAs, which contain sequences for the mature 18S, 5.8S, and 25S rRNAs, two external transcribed regions, and two internal transcribed spacers (ITSs). An initial large 90S particle is subsequently separated into precursors of the large 60S and the small 40S ribosomal subunits.

The process of protein synthesis occurs in 4 basic steps: initiation, elongation, termination, and ribosome recycling. P. murina had many genes associated with these processes, as well as ribosomal biogenesis, that were downregulated in response to anidulafungin treatment, with the exception of translation initiation factors.

### (vi) 40S subunit.

Ribosomal protein S6 (Rps6) of the 40S subunit of the eukaryote ribosome, with homology to EMR09837.1, was first identified as the only ribosomal phosphoprotein in rapidly growing cells, and the S6 phosphorylation state has been linked to translational activity (58). Rps6 phosphorylation is sensitive to nutrients, hormones, growth factors, and a variety of stress conditions. During maturation of the 40S subunit, a final rRNA processing step of 20S pre-rRNA into mature 18S rRNA occurs by Nob1p, an endonuclease. Prior to this cleavage, the ribosomal protein 3 (Rps3) (EMR10537.1) must provide a critical structural reorganization step for the process to proceed (59). Budding yeast expresses 2 proteins that localize to the nucleolus and are associated with efficient processing of pre-rRNA: Fcf1p, of which P. murina has a homolog (EMR08677), and Fcf2p (57). Together the genes coding for these proteins facilitate the early steps leading to the synthesis of 18S rRNA.

Ribosomal protein S5 (rpS5) of eukaryotes (EMR10517.1) is the counterpart of prokaryotic rpS7. There is much conservation among sequences from the 2 different kingdoms, especially at the central/C-terminal region, indicating it serves an important function (60). These proteins form part of the exit site on the 30S/40S ribosomal subunits and help to form the mRNA exit channel. Mutations in rpS7/S5 lead to dysregulated cell function and perturbation of the translation process. Mutations specific to the N terminus were predicted to affect the ability of the 40S subunits to recruit initiation factors eIF3p and eIF2p, resulting in inefficiencies of translation.

### (vii) 60S subunit.

The P. murina homolog to the rRNA processing protein, Rrp17p (EMR11496.1), is an exonuclease required for 5′-end processing of pre-60S rRNA and is part of the yeast nuclear pore complexes (61). It is associated with the late 60S preribosomes and was shown to associate with precursors to 5.8S and 25S rRNAs, providing exonuclease digestion to produce these products from the preribosome 60S. Deletion of the gene coding for this protein results in lethality.

### (viii) Elongation.

In fission yeast, the homolog to EMR11019.1, an elongation factor of the Elf1 family, is predicted to aid in the maintenance of proper chromatin structure in regions of active transcription from the RNA polymerase II promoter and located in the nucleus (83). This association is partially dependent on Spt4p and Spt6p, which together regulate transcription elongation.

### (ix) Termination and recycling.

Termination and recycling are processes essential to release of the completed polypeptide and in turn recycle the 40S and 60S subunits for subsequent rounds of translation. In yeast, the 40S recycling factors include Tma20p, Tma22p, and Tma64p, of which the homolog to Tma20p (EMR09557.1) was downregulated in the treated organisms.

Together, the downregulation of genes necessary for ribosomal biogenesis indicates a dysregulation of translation leading to reduced protein synthesis. In both fission and baker's yeast, a reduction of ribosome function and levels due to nutrient depletion leads to flocculation of the cells with subsequent initiation of sexual reproduction (62). It was suggested that yeast cells could sense these environmental changes and that sexual reproduction was a response to unhealthy environments. We posit the same could be happening in the anidulafungin-treated P. murina, which senses an inhospitable environment resulting from the drug treatment and in turn decreases ribogenesis and protein synthesis, leading to attempts to undergo sexual reproduction.

### (x) Cellular homeostasis/cell wall integrity.

Sho1p (EMR10852.1) in yeast was long considered an osmosensor for the HOG (high osmolarity glycerol) MAP kinase pathway, which controls glycerol production and regulates the osmotic state of the cell and thus could regulate cell fusion (63). Downregulation of Sho1p by Fus1p was hypothesized to lower the internal osmolarity of cells during fusion during the mating process, inhibiting cell lysis and death (64). More recent studies have demonstrated its importance in cytokinesis, where it was shown to be essential for correct cell-to-cell separation (65). Prior to cytokinesis, Sho1p sequentially assembles with Hof1p, Inn1p, and Cyk3p, and this assembly may connect the membrane with the actin-myosin ring. This activity does not appear to be linked to its role as an osmosensor.

One sequence, EMR09915.1, shared limited homology to the osmotic tolerance-associated gene of S. cerevisiae, *Hal9*, and a predicted transcription factor, zinc finger (zf)-fungal binuclear type. In yeast, *Hal9p* deletion was associated with a decline in sodium and lithium tolerance, while deletion of the homolog in Candida glabrata resulted in the inability to survive in an acidic environment (84). Whether this bears any relation to such functions in Pneumocystis cannot be discerned at this time.

Degradation of proteins is an essential activity that maintains cellular homeostasis. This function is by and large the role of the proteasome, outside the lysosomal protein degradation in eukaryotes. Proteasome assembly is complex and not entirely defined. However, some cofactors have been identified that facilitate proper assembly and maturation, which include Ump1p (ubiquitin-mediated proteolysis) (EMR08979.1) (66). Ump1p is a rapidly degraded cofactor (∼17 kDa) that is necessary for the efficient biogenesis of the 20S proteasome (core protein), which is the catalytic core particle of the 26S proteasome. Of note, 2 members of the Hsp70 family in yeast, *ssa1* and *ssa2*, assist in CP assembly—perhaps by a stabilization of intermediates—and were upregulated (Table 1) (67), while *Ump1* was downregulated in response to anidulafungin, suggesting a dysregulation of the stress response.

Stress-related signals included homologs to yeast *yke2* (EMR11819.1), which is a component of the heterohexameric Gim complex (GimC) (also known as prefoldin), which binds and stabilizes unfolded target polypeptides, conveying them to chaperonins for completion of folding (68). The Gim complex specifically targets the formation of properly folded and functional α- and β-tubulins (69). Deletion of the genes coding for these proteins causes microtubule defects, which play important roles in chromosome segregation in mitosis and meiosis and organelle positioning and transport functions. Thus, their downregulation could result in aberrant replication and intracellular morphology in the anidulafungin-treated P. murina.

### Downregulation of genes 8 days after treatment cessation.

After 8 days without anidulafungin treatment and with continued immunosuppression, only 5 genes were significantly downregulated, while no genes were significantly upregulated (see Table S1 in the supplemental material). Genes with homologs included the *Meu14* genes, which were also observed to be downregulated in the untreated P. murina compared to the treated P. murina (day 0) (*pil1* and *lsp1*). Likewise, the hypothetical proteins PNEG_02319 and -01236 were also downregulated compared to in the treated mice at day 0, perhaps indicating that the effects of anidulafungin were wearing off after 8 days of cessation of treatment. The EMR08989.1 sequence shared homology with the gene coding for peptidyl-tRNA hydrolase ICT1, mitochondrial-like, from plants, mammals, and zebrafish, but not with any fungal gene.

### Validation of expression of selected genes.

**FIG 4:**
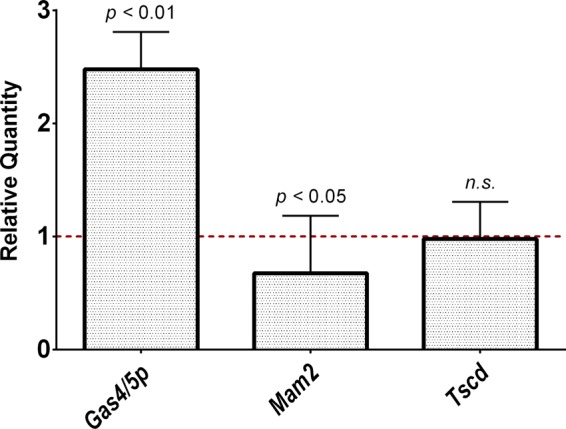
Validation of RNA-seq mRNA expression data by real-time quantitative PCR. Total RNA was extracted from P. murina-infected mice treated with anidulafungin for 2 weeks and nontreated infected mice. cDNA was synthesized from total RNA and used as the template for RT-qPCR. The relative quantity of Gas4/5p was upregulated, while Mam2 has decreased expression, validating RNA-seq data. *Tscd* is shown as a control representing a gene with no gene expression regulation. The red dotted line indicates baseline expression in untreated mice. Error bars indicate standard error of the mean (SEM). *n.s.*, not significant.

To confirm the results of the RNA-seq expression profiles, transcript abundance of the *Gas4*/*5p*, *Mam2*, and 3-ketodihydrosphingosine reductase (*TscD*) genes was validated by reverse transcription-quantitative PCR (RT-qPCR). *Gas4/5p* was upregulated in anidulafungin-treated mice (Table 1). RT-qPCR experiments validated increased expression of *Gas4*/*5p* (Fig. 4; *P* < 0.01). This supports the hypothesis that Gas4/5p is attempting to modulate the formation of ascus cell membrane maturation as P. murina tries to enter sexual reproduction but is unable to do so. Expression of *Mam2* in anidulafungin-treated mice displayed a downregulation in the RNA-seq expression profile. This downregulation was validated in RT-qPCR experiments (Fig. 4; *P* < 0.05). *TscD* (tubulin-specific chaperone D) was not significantly up- or downregulated in RNA-seq data and therefore was used as a control for minimal gene expression change. *TscD* showed a similar expression profile in RT-qPCR data as RNA-seq data, in that no gene expression changes were detected (fold change of 1.0). Collectively, these results validate gene expression changes detected by RNA-seq.

### Conclusions.

It is clear from the results of this RNA-seq study that anidulafungin exerted a plethora of effects on P. murina, outside of targeting BG synthesis. The interruption of the synthesis of BG resulted in the lack of production of new asci, which would be expected, but the apparent degradation of existing asci would be hard to explain by a halt in synthesis of BG. The almost immediate effect on the degradation of the asci was shown in early reports by electron microscopic studies (70). Additional information from this study, including the upregulation of glucanases and other degradative enzymes, leads us to hypothesize that the disappearance of existing asci was due to activation of these enzymes, a heretofore unreported aspect of anidulafungin treatment. Measurement of the BG in mouse lungs showed a dramatic reduction in the lungs of treated mice, lending additional support to this hypothesis. Upregulation of other enzymes involved in cell remodeling suggests that the P. murina cells are reacting in a concerted way to the cell wall perturbations effected by the echinocandin.

A striking result was the signature of upregulated genes associated with sexual reproduction. We interpret this as a reaction to the metabolic stress placed on the P. murina population. Many microbes react to a stressful environment by undergoing sexual reproduction to form spores of some kind that resist the stress or disperse the next generation to a more favorable location. In this situation, the treated P. murina cell is compromised because it must form an ascus to produce the ascospores but it cannot synthesize the BG to enable this effort. Previous studies in our lab showed that the forms of P. murina remaining after echinocandin treatment were unable to proliferate and also exhibited aberrant morphology (3). This infers that a complete cell cycle block resulted from the treatment, and perhaps in G_1_, as reported for other fungi treated with echinocandins (4). While we had previously assumed that asexual replication in Pneumocystis species was not linked to the sexual cycle (71), the data presented here indicate this may not be the case. The signal in the downregulated genes was more diverse but seemed to reflect a generalized dysregulation of the treated fungi. We observed upregulation of several genes related to meiosis and a reduction in expression of Mam2 and Prm1 homologs, both of which are also related to mating. This profile indicates that meiosis and sexual reproduction were not in fact occurring, although the fungi were exerting efforts to do so. The remaining downregulated genes underscore organisms under stress, with reduced ribosome biogenesis and protein translation, mitochondrial collapse, and reduction in expression of the Msg genes, which code for the dominant surface antigens. A search for orthologs in the genome of P. murina related to genes in other fungi, specifically C. albicans *cas5, swi4, swi6, mig1, msn4, mig2*, and *msn2*, associated with the perturbations induced by echinocandins, did not reveal any convincing homologies, suggesting that Pneumocystis may use different pathways, or there is little homology to these particular genes.

It is important to note that a decrease in ribosome biogenesis and protein synthesis has been linked to flocculation and initiation of sexual reproduction of yeast in nutrient-depleted environments, lending credence to our underlying hypothesis that anidulafungin creates an inhospitable environment, driving P. murina to decrease protein synthesis, leading to attempts to undergo sexual reproduction.

This study provides new insights and avenues for further study on the effects of the echinocandins on Pneumocystis and potential new targets for combination therapies. It has also revealed new information on the metabolism of these pathogenic fungi and their responses to stress.

## MATERIALS AND METHODS

### Compounds.

Eraxis (anidulafungin for injection; Vicuron Pharmaceuticals, Inc.) was used at 100 mg/vial reconstituted in sterile water. Dexamethasone (Vedco, Inc., St. Louis, MO) was administered at 4 mg/liter to acidified drinking water (sulfuric acid at 1 ml/liter).

### Study 1: anidulafungin treatment of P. murina-infected mice.

Thirty-six male C3H/HeN mice (20 g) were purchased from the Charles River Laboratories (Raleigh, NC) and shipped in filtered Sew-Easy shipping crates that were UV irradiated prior to placement of mice inside the box and then sewn shut. Upon receipt in the Cincinnati Veterinary Medicine Unit (VMU) facilities (Cincinnati VAMC, Cincinnati, OH), mice were uncrated under sterile conditions by transfer under laminar flow into microisolator cages with filter tops (Reemay 2033, 15-ml thickness, 250-cfm airflow, 15-μm exclusion; Kavon Filter Products Co., Farmingdale, NJ). Once in the VMU, the mice received sterilized mouse chow (autoclavable rodent diet 5010; LabDiet, St. Louis, MO) and acidified sterile water to reduce bacterial blooms from indigenous microbes. After 2 days of acclimation, the mice were immunosuppressed by administration of 4 μg/liter of dexamethasone in the acidified water. It was expected that the mice received from the vendor would not harbor P. murina due to the barrier housing. All mouse housing and handling conformed to IACUC-approved protocols.

### (i) Experimental model.

All 36 immunosuppressed mice were exposed to P. murina-infected mice for 2 weeks to transmit the infection (a process termed “seeding,” which is the most “natural” method of infection) (72). After a period of 5 weeks with continued immunosuppression (to permit the infection to reach moderate organism burdens), 30 of the 36 mice were started on 1 mg/kg anidulafungin for 2 weeks, delivered by intraperitoneal injection. Anidulafungin was used as the echinocandin of choice, as we had shown its effects were similar to caspofungin and it was selected for further analysis in our previous study; micafungin was the least efficacious of the 3 drugs tested (3). After 2 weeks of anidulafungin treatment, 6 treated mice and the 6 untreated mice were sacrificed, lungs were weighed, and a small piece of lung was taken from the left lobe, weighed, and processed for organism enumeration (3). The remaining lung was snap-frozen in liquid nitrogen to retain the integrity of the mRNA for RNA-seq analysis. The remaining 24 mice were taken off anidulafungin but continued on immunosuppression. At days 2, 4, 6, and 8, 6 mice that were taken off anidulafungin were sacrificed and lungs processed as described above.

### (ii) Enumeration of “trophic” forms and asci.

The left lung lobe piece was homogenized, and slides were stained with cresyl echt violet (CEV), which selectively stains the asci, and a rapid version of the Wright-Giemsa stain (Leuko-stat; Fisher Scientific) to enumerate the nuclei of all the non-BG-expressing life cycle stages remaining after anidulafungin treatment, collectively referred to as “trophic” forms (3). The microscopic counts were log transformed, and values were compared by the one-way analysis of variance (ANOVA) followed by Newman-Keuls multiple-comparison posttest using GraphPad software for Science v7 (GraphPad, San Diego, CA). Significance was accepted when the *P* value was <0.05. The limit of detection by this method is 1.75 × 10^4^ (log_10_ 4.24/lung).

### Study 2: anidulafungin treatment of P. murina-infected mice for follow-up BG studies.

To follow up on the RNA-seq observations of potential digestion of BG by hydrolases and glucanases, a small study was conducted to supply additional lung homogenates for analysis. Five mice were in the anidulafungin-treated group, and 4 were in the untreated group. Infection and treatment with 1 mg/kg anidulafungin were conducted as described above. After 2 weeks of treatment, the mice were sacrificed and lungs were removed for organism enumeration (3). Log_10_ values for nuclei and asci were 7.46 ± 0.1283 nuclei and 7.10 ± 0.1541 asci in the untreated mice and 7.30 ± 0.0581 nuclei and 4.42 ± 0.00 asci in the treated mice. The differences between the groups were significant for nuclei (*P* = 0.0439) and asci (*P* < 0.001). Statistical comparison of the organism burdens in study 1 versus study 2 revealed a single significant difference. Counts of nuclei in the untreated mice in the second study were lower than those in the first (*P* = 0.0067). This is not unexpected as organism burdens to vary from one animal experiment to another and did not influence the BG studies as there was no significant difference in counts of asci between the studies (see Fig. S2 in the supplemental material).

### Ethics statement.

The animal protocols used for this study were reviewed and approved by the University of Cincinnati's IACUC committee and the Cincinnati Veterans Affairs Medical Center IACUC: protocols UC 12-05-03-01 and ACORP15-02-25-01, respectively. Both committees adhere to the 8th edition of the *Guide for the Care and Use of Laboratory Animals*, and both are AAALAC accredited.

### RNA extraction and treatment.

All the following steps were carried out at the University of Cincinnati Genomics, Epigenomics and Sequencing Core, Department of Environmental Health, University of Cincinnati, Cincinnati, OH (http://eh.uc.edu/genomics/). The snap-frozen lung tissues were treated with RNAlater solution (Thermo Fisher, Grand Island, NY) to stabilize the RNA. Tissue was homogenized in 1 ml lysis/binding buffer from the mirVana microRNA (miRNA) isolation kit (Thermo Fisher, Grand Island, NY) by using a Precellys 24 homogenizer. The total RNA extraction protocol was conducted according to the vendor's protocol, and the RNA was eluted with 100 μl elution buffer.

The Ribo-Zero Gold (Human/Mouse/Rat) and (Yeast) kits (Illumina, San Diego, CA) in a 1:1 ratio were used to deplete rRNA before library preparation for RNA-seq. The kits efficiently remove the highly abundant cytoplasmic and mitochondrial rRNAs isolated from mouse cells and Pneumocystis cells and enrich the mRNA, poly(A) or non-poly(A) long noncoding RNA (lncRNA) and other known/unknown RNAs. The core used the WaferGen (Pleasanton, CA) Apollo 324 system and ran Ribo-Depletion script for the automated targeted RNA depletion with 100 ng to 1 μg total RNA as input. We combined (1:1 ratio) the Ribo-Zero Gold (Human/Mouse/Rat) kit with the Ribo-Zero Gold (Yeast) kit to remove rRNA from both the mouse and yeast (Pneumocystis) in the mixed samples.

A commercially available magnetic mRNA isolation kit was used for the poly(A) RNA [including mRNA and poly(A) lncRNA] purification. A total of 0.1 to 1 μg total RNA or depleted RNA was used as the input. The core used the Apollo 324 system (WaferGen, Fremont, CA) and ran PrepX PolyA script for the automated poly(A) RNA isolation.

### Automated RNA-seq library preparation.

Using the PrepX mRNA library kit (WaferGen) and Apollo 324 NGS automated library prep system, the depleted or isolated RNA was RNase III fragmented, adaptor ligated, and Superscript III reverse transcriptase (Lifetech, Grand Island, NY) converted into cDNA, followed by automated purification using Agencourt AMPure XP beads (Beckman Coulter, Indianapolis, IN). The targeted cDNA fragment is around 200 bp. Five hundred nanograms total RNA was used as input, and 13 cycles of PCR were performed to introduce the index and amplify the library.

To check the quality and yield of the purified library, 1 μl of the library was analyzed by Bioanalyzer (Agilent, Santa Clara, CA) using a DNA high-sensitivity chip. To accurately quantify the library concentration for the clustering, the library was 1:10^4^ diluted in dilution buffer (10 mM Tris-HCl [pH 8.0] with 0.05% Tween 20), and qPCR was measured by the Kapa library quantification kit (Kapabiosystem, Woburn, MA) using ABI's 9700HT real-time PCR system (Thermo Fisher).

### RNA-seq analysis and transcript annotation.

Raw shotgun reads were trimmed from both ends by 3 nucleotides to remove adaptor and low-quality sequences, resulting in 45-bp reads. RNA-seq data for the mouse and Pneumocystis were analyzed using AltAnalyze (73), which employs Kallisto for ultrafast mapping of transcripts (74). Gene expression was computed as log_2_-transformed transcripts per million (TPM). Significance of differential expression was set to a fold change of >1.5 and an adjusted *P* value of <0.05. *P* values were adjusted for multiple-hypothesis testing using the Benjamini-Hochberg correction.

### (i) Expression validation.

Genes of interest in P. murina to be validated were identified from analysis of data obtained from RNA-seq. Validations included upregulated, downregulated, and nonregulated genes. Oligonucleotide primers to the genes of interest were designed based on sequences from NCBI/GenBank. Primers were designed to span an intron junction to prevent amplification of genomic DNA. Reactions with primers designed to P. murina were run with primers to the Pneumocystis *Tscd*, which corresponds to PNEG_02941, which shows a BLAST result for tubulin-specific chaperone D. The relative quantity of *Tscd* in treated day 0 versus untreated is 0.978175071 (*P* = 0.944101), with a fold change of 1.00000683 and an adjusted *P* value of 1. Each set of primers also had a negative control that contained no cDNA template.

cDNA from the same mice used for RNA-seq were used as the templates. RNA was isolated from the lungs of these mice using TRIzol reagent (Life Technologies, Grand Island, NY) per the manufacturer's protocol. cDNA was immediately synthesized from the extracted RNA using SuperScript IV VILO master mix with ezDNase enzyme (Invitrogen, Grand Island, NY) per the manufacturer's protocol. The remaining RNA and the freshly synthesized cDNA were stored at −80°C.

Real-time PCR was performed in an Applied Biosystems 7500 fast real-time PCR system (Life Technologies, Grand Island, NY). The reactions were performed in triplicate in a final volume of 20 μl containing 1× PowerUp SYBR green master mix (Life Technologies, Grand Island, NY) and 500 nM each primer pair (Table 3). The reaction mixtures were initially incubated at 50°C for 2 min and 95°C for 2 min, followed by 40 cycles of 15 s at 95°C, 59°C for 15 s, and 72°C for 1 min. Fluorescence data were captured during the 72°C step. Disassociation curves were also calculated for the reactions to determine the melting temperature (*T_m_*) of the products. The threshold cycle (Δ*C_T_*) value between the validation gene and the thymidylate synthase (TS) gene was calculated by subtracting the average *C_T_* value of the single-copy reference gene from the average *C_T_* value of the validation gene. ΔΔ*C_T_* values were calculated by subtracting the Δ*C_T_* value of the reference sample from the Δ*C_T_* value of the experimental sample. Relative quantity is shown as 2^−ΔΔ*CT*^.

**TABLE 3:** Primers used for RT-PCR

Gene target	Primer	Primer sequence 5′→3′	Accession no.
*Gas4/5p*	Forward	TCATGGTGTTCTCCTTCCTCAT	XM_007873707
	Reverse	GGCCTTGGGACTGCTCTATT	
*Mam2*	Forward	TGGCACTTGTCCTTATGTTGAC	XM_007877017
	Reverse	AGGTGCCTGACAAATAAGCA	
*Tscd*	Forward	CCACACGAAGCATCATGGATTC	XM_007876794
	Reverse	GCTGGATGTAGAGCCACATC	

### (ii) Homology assessments.

The BLASTp suite was used to assign initial gene homologies with a *post hoc* analysis using SmartBLAST (National Library of Medicine, National Center for Biotechnology Information), which is available from https://blast.ncbi.nlm.nih.gov/smartblast/. SmartBLAST is a new tool from NCBI that will not only identify the closest-matching sequences but will also identify the closest matches of high-quality sequences from model organisms. The tool then constructs a phylogenetic tree via multiple-sequence alignment from the top 5 closest matches, allowing quick visual assessments. Though SmartBLAST was used to assign homology to mostly S. pombe and S. cerevisiae sequences because of their phylogenetic relationship to Pneumocystis and because of the knowledge base associated with these yeasts, it is important to note that higher levels of homology were often achieved with other species, such as Saiotella, which is in the same clade as the Pneumocystis species.

### Measurement of β-1,3-d-glucan in the lungs.

The total β-1,3-d-glucan contents in anidulafungin (1-mg/kg)-treated and untreated mouse lungs were measured using the Glucatell (Associates of Cape Cod, Inc., East Falmouth, MA) endpoint assay according to the vendor's instructions. Aliquots were resuspended in 1 ml of 1 M sodium hydroxide and shaken at room temperature for 1 h. Samples were prepared as 10-fold dilutions in pyrogen-free water in test tubes certified to be free of glucan (Associates of Cape Cod). Glucan standards were prepared in the same manner according to vendor instructions, The β-1,3-d-glucan content was measured using a linear regression curve of standards from 25 to 200 pg/ml. The correlation coefficient for the studies presented was 0.992, within the acceptable range suggested by the vendor (>0.999). Glucan content was expressed as picograms per milliliter. An unpaired *t* test was used to assess significance (GraphPad Instat v.6).

### Fluorescent labeling of the major surface glycoprotein (Msg) and β-1,3-d-glucan of P. murina-infected mice.

P. murina-infected lung homogenates from untreated and anidulafungin-treated mice from study 2 were treated with ammonium chloride to remove red blood cells. Samples were washed, resuspended in phosphate-buffered saline (PBS), and placed on slides to air dry. Samples were fixed and permeabilized in acetone (Sigma-Aldrich, St. Louis, MO) for 1 min. Nonspecific binding sites were blocked using 1% bovine serum albumin (BSA) (Fisher Scientific, Fair Lawn, NJ) in PBS. Samples were then incubated with anti-β-1,3-d-glucan monoclonal antibody (MAb) (Biosupplies Australia, Victoria, Australia) and anti-Msg polyclonal antisera (8) at 4°C for 1 h. The β-1,3-d-glucan MAb does not cross-react with 1,4-α-glucan or (1→3, 1→4)-β-glucan. After washing, Alexa Fluor 488-conjugated goat anti-rabbit and Alexa Fluor 594-conjugated goat anti-mouse IgGs (Invitrogen, Waltham, MA) in 1% BSA in PBS were added as secondary antibodies and incubated at 4°C for 1 h. Samples were imaged under epifluorescence using an Olympus BX51 microscope and Olympus DP72-BSW camera and software. An Olympus U-RFL-T mercury lamp was used with appropriate filters for detection of Alexa Fluor 468 (excitation, 490 nm; emission, 525 nm) and Alexa Fluor 568 (excitation, 578 nm; emission, 603 nm).

### Accession number(s).

RNA-seq files have been deposited in NCBI under accession no. PRJNA433993.

## Supplementary Material

Supplemental material
