# Supplementary material for: Gene Expression of Pneumocystis murina after Treatment with Anidulafungin Results in Strong Signals for Sexual Reproduction, Cell Wall Integrity, and Cell Cycle Arrest, Indicating a Requirement for Ascus Formation for Proliferation
Source: Antimicrob Agents Chemother. 2018 Apr 26;62(5):e02513-17. doi: 10.1128/AAC.02513-17 (PMC5923105; doi:10.1128/AAC.02513-17)
Supplement: Supplemental material [file supp_62_5_e02513-17__index.html]

Supplemental material 

# Gene Expression of Pneumocystis murina after Treatment with Anidulafungin Results in Strong Signals for Sexual Reproduction, Cell Wall Integrity, and Cell Cycle Arrest, Indicating a Requirement for Ascus Formation for Proliferation

## Supplemental material

- Supplemental file 1 -

  Supplemental Table S1 and Figures S1 and S2

  PDF, 101K
