# Supplementary material for: Gene Expression of Pneumocystis murina after Treatment with Anidulafungin Results in Strong Signals for Sexual Reproduction, Cell Wall Integrity, and Cell Cycle Arrest, Indicating a Requirement for Ascus Formation for Proliferation
Source: Antimicrob Agents Chemother. 2018 Apr 26;62(5):e02513-17. doi: 10.1128/AAC.02513-17 (PMC5923105; doi:10.1128/AAC.02513-17)
Supplement: Supplemental material [file AAC.02513-17_zac005187107s1.pdf]

Supplemental Table 1. Genes down regulated in *P.murina* 8 days after anidulafungin cessation vs 2 days post cessation

GenBank ID	Gene description	UnTx[1]	Tx0d[2]	FC (Untx/ Tx0d)[3]	E-Value[4]	Identity (Organism)[5]
EMR09370.1	Hypothetical protein PNEG_02319	7503.823	2041.254	3.676	2e-92	60%/HP T552 02875/Pc
EMR10527.1	hypothetical protein PNEG_01236	119.498	34.652	3.448	1e-115	92% T552 04055/Pc
EMR08655.1	Sphingolipid long chain base-responsive Pil1	143.502	48.157	2.980	3e-129	64%/Sc
EMR09455.1	Sphingolipid long chain base-responsive LSP1	217.555	85.905	2.533	2e-34	33%/Sp
EMR08989.1	Peptidyl-tRNA hydrolase domain	440.360	200.931	2.192	7e-21	40%/Gm

[1]UnTx – *P. murina* extracted from infected and untreated mice. Expression values are TPM.

[2]Tx0d – *P. murina* extracted from infected mice treated with anidulafungin for 2 weeks. Expression values are TPM.

[3]FC – Fold change in *P. murina* gene expression between infected untreated mice and those treated with anidulafungin for 2 weeks.

[4]E-value of sequence homology to the known genes as reported by NCBI SMARTBLAST tool.

[5]Sequence identity (percentage) to the known genes and organisms. Abbreviations: Gm (*Glycine max*) Pc (*Pneumocystis carinii*); Pj (*Pneumocystis jirovecii*); Sc (*Saccharomyces cerevisiae*); Sp (*Schizosaccharomyces pombe*).

* Hypothetical Proteins (HP) organism and identity are for the closest species that were not *P.murina* in origin

Supplemental Figure 1. Aberrant morphology of anidulafungin-treated *P. murina.*

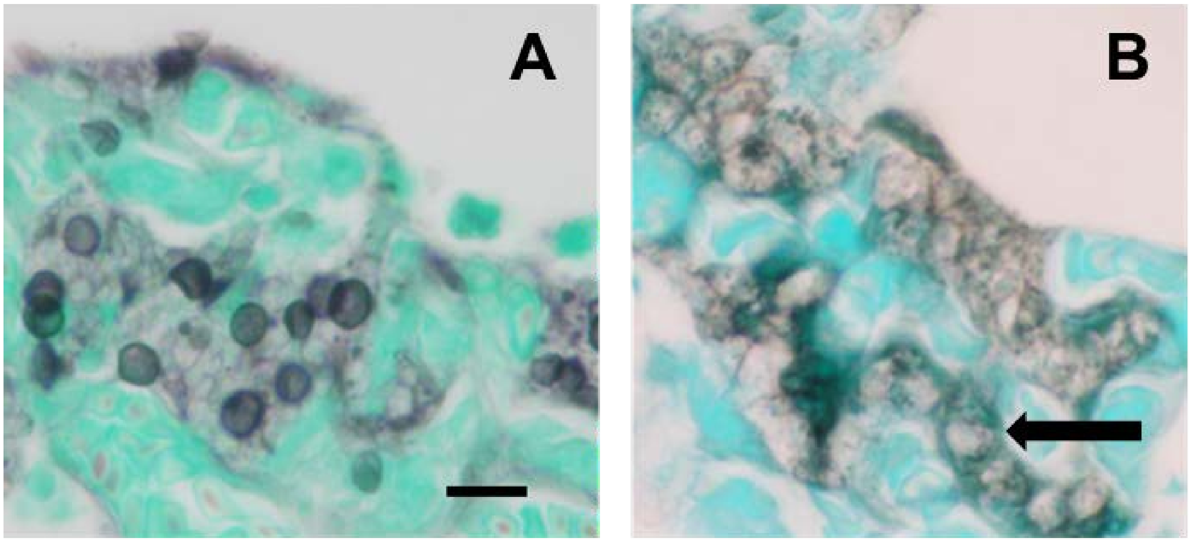

Supplemental Figure 1. Aberrant morphology of anidulafungin-treated *P. murina.*

Grocott's methenamine silver-stained sections of lungs from: untreated mice (Panel A); and from mice treated with 1 mg/kg anidulafungin (Panel B) (archival material from reference 3). 1,250× magnification; bar is approximately 10µm. Note the circumscribed asci morphology and the dark methenamine silver staining in the first panel and the lack of typical asci and loss of silver staining in the second panel.

Supplemental Figure 2. Comparison of organism burdens (nuclei and asci) between Study 1 and Study 2.

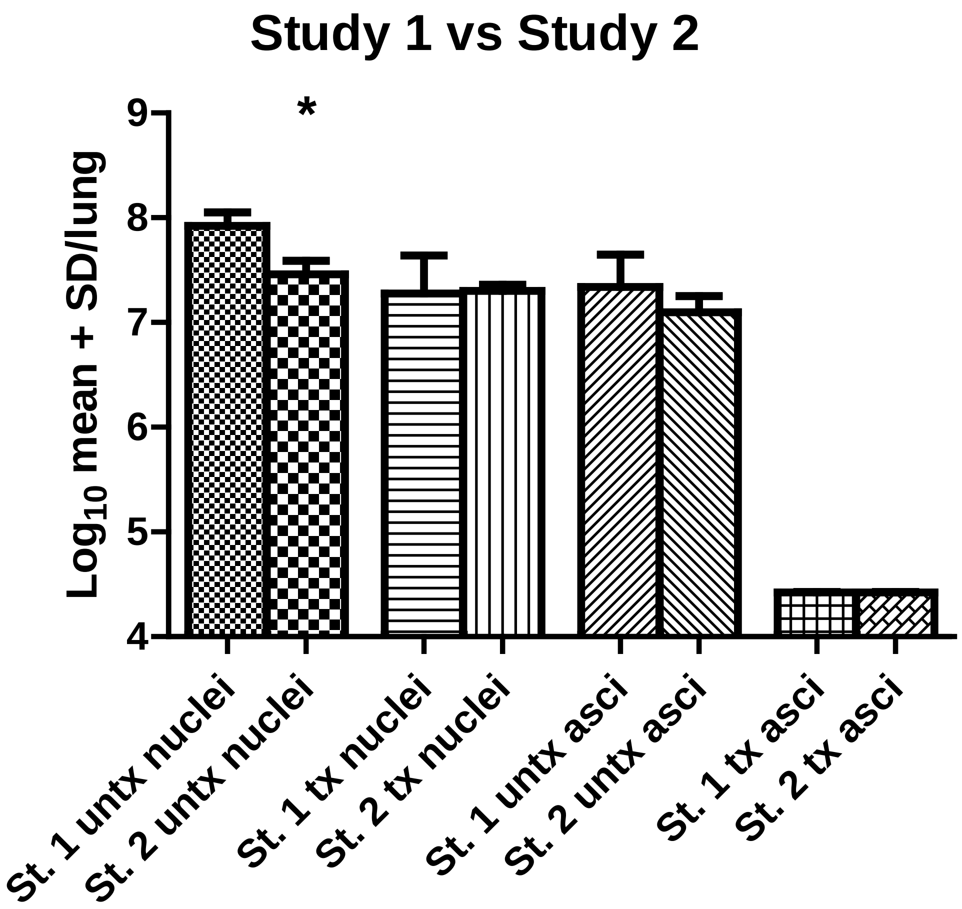

***Previous study untx nuclei vs new study; p value 0.0067**

Supplemental Figure 2. Comparison of organism burdens (nuclei and asci) between Study 1 and Study 2.

Total nuclei and asci counts from lung homogenates of untreated (untx) and anidulafungin treated (tx) mice were analyzed for significant differences using Unpaired t test for each pair. The only significant difference was identified in total nuclei counts between the untreated and treated mice (*); P=0.0005
